# A novel agent for myeloma causing toxic keratopathy, belantamab mafodotin: a case report and literature review

**DOI:** 10.5935/0004-2749.2021-0358

**Published:** 2022-10-19

**Authors:** Rengin Aslıhan Kurt, Deniz Gören, Sehnaz Karadeniz, Mutlu Arat, Afsun Sahin

**Affiliations:** 1 Department of Ophthalmology, Baskent University Istanbul Hospital, Istanbul, Turkey; 2 Department of Hematology, Sisli Florence Nightingale Hospital, Istanbul, Turkey; 3 Department of Ophthalmology, Sisli Florence Nightingale Hospital, Istanbul, Turkey; 4 Department of Ophthalmology, Koc University Medical School, Istanbul, Turkey

**Keywords:** Multiple myeloma, Belantamab mafodotin, Confocal microscopy, Cornea, Drug-related side effects and adverse reactions, Mieloma múltiplo, Belantamabe mafodotina, Microscopia confocal, Córnea, Efeitos colaterais e reações adversas relacionados a medicamentos

## Abstract

A 60-year-old-male with refractory relapsed multiple myeloma presented with redness, pain, foreign body sensation, and blurred vision in both eyes that gradually increased after his third belantamab mafotodin infusion. Biomicroscopy revealed bilateral microcyst-like epithelial changes and epithelial crystal-like deposits, whereas in vivo confocal microscopy revealed intraepithelial and subepithelial hyperreflective deposits in corneal epithelium. Belantamab mafodotin therapy was discontinued for seven weeks due to corneal toxicity, which cleared progressively. We aim to demonstrate belantamab mafodotin-related corneal toxicity that may be detected using slit lamp and in vivo confocal biomicroscopy.

## Abstract (pt)

Um homem de 60 anos, diagnosticado com mieloma múltiplo recidivante refratário, apresentou vermelhidão, dor, sensação de corpo estranho e visão turva em ambos os olhos, aumentando gradualmente após sua terceira infusão de belantamabe mafodotina. À biomicroscopia, foram observadas alterações epiteliais bilaterais semelhantes a microcistos e depósitos epiteliais semelhantes a cristais. A microscopia confocal *in vivo* revelou depósitos hiper-refletivos intraepiteliais e subepiteliais na córnea. Devido à toxicidade corneana, a terapia com belantamabe mafodotina foi interrompida por sete semanas e a toxicidade foi gradualmente resolvida. Nosso objetivo é demonstrar os achados à biomicroscopia confocal in vivo e à lâmpada de fenda da toxicidade corneana relacionada ao belantamabe mafodotina.

## INTRODUCTION

Multiple myeloma (MM) is a hematologic malignancy characterized by the proliferation of clonal plasma cells in the bone marrow or extramedullary areas^(1^,^2)^. Despite recent advances in management, the prognosis remains poor, and MM is still accepted as an incurable entity^(3)^. Relapse or refractory disease, defined as resistance to standard treatment protocols, has a particularly unfavorable prognosis^(2^,^3)^. Within two decades, myeloma survival improved, particularly with more approval of novel agents^(4)^.

Belantamab mafodotin (Blenrep, GlaxoSmithKline, St. Louis, MO, USA) is a monoclonal antibody-drug conjugate (ADC) that targets B-cell maturation antigen (BCMA) on malignant plasma cells^(5)^. It is a humanized IgG1κ monoclonal antibody conjugated with monomethyl auristatin F (MMAF), a cytotoxic agent. When the ADC binds to BCMA on the myeloma cell surface, the process of cytotoxic microtubule inhibition begins. Belantamab mafodotin (BM) is approved both in the USA and Europe in August 2020, according to the results of the multinational DREAMM-2 trial, and is used for the treatment of relapsed or refractory MM, who have received at least four lines of therapy^(6)^.

Corneal toxicity is one of the most frequent side effects of BM treatment. According to the DREAMM-2 study results, 72% of the cases had signs of corneal toxicity like microcystic epithelial changes (MECs)^(6)^. We present a case of BM corneal toxicity and emphasize the importance of in vivo confocal biomicroscopy (IVCM) in both diagnosis and follow-up.

## CASE REPORT

**Figure 1:**
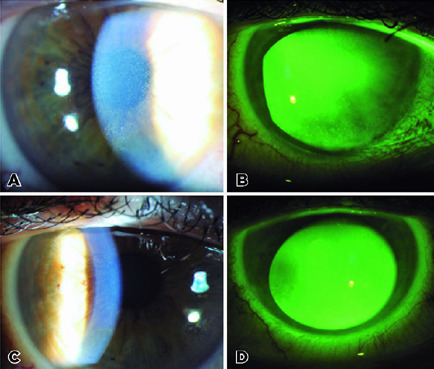
Slit lamp biomicroscopic image showing numerous intraepithelial cyst-like crystal deposits in the cornea epithelium (A) and punctate keratopathy (B) at the time of diagnosis. Significant reduction in the intraepithelial deposits (C) and resolution of punctate keratopathy can be seen after the cessation of the therapy.

**Figure 2:**
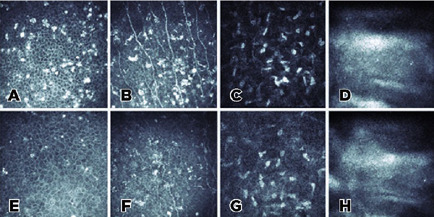
*In vivo* confocal images of the cornea at the time of diagnosis due to BM toxicity (A-D) and after 7 weeks cessation of therapy (E-H). Notably, there was a significant reduction in corneal deposits after cessation of BM therapy.

A 60-year-old-male patient complained of redness, pain, burning, stinging, foreign body sensation, and blurred vision in both eyes. He was diagnosed with MM nine years ago and underwent autologous hematopoietic stem cell transplantation. He underwent an allogeneic stem cell transplantation from an unrelated matched donor due to a refractory disease course. However, he experienced a relapse four years ago. He still had a progressive disease course after three lines of chemotherapy. Therefore, a novel agent, BM, 2.5 mg/kg was started. After the initiation of BM, an in-house ophthalmologist began to evaluate the patient’s ocular status periodically as required. He had received his third BM infusion three days before his referral to our department. On examination, the best corrected visual acuities were 20/25 in the right eye and 20/40 in the left eye; a decline from a baseline of 2 lines. On biomicroscopic examination, epithelial crystal-like deposits were seen on both corneas with a left predominance (Figure 1A). Corneal fluorescein staining showed diffuse punctate epitheliopathy in both eyes (Figure 1B). IVCM showed intraepithelial and subepithelial hyperreflective deposits, mainly accumulated within the wing and basal cells (Figure 2A). Mature activated dendritic cells were increased in the subbasal area. Subbasal corneal nerve plexus loss was significant (Figure 2B). Keratocyte activity in corneal stroma was normal, as were endothelial cells (Figures 2 C, D). The right eye’s intraocular pressure was 15 mmHg, whereas the left eye was 17 mmHg. A dilated fundus examination revealed a normal optic disc appearance. In both eyes, the macula and peripheral retain areas were within normal limits.

With all these findings the patient was diagnosed with moderate BM corneal toxicity, and the treatment was discontinued. Artificial eye drops and topical loteprednol etobonate eye drops were prescribed twice daily. The patient was admitted to our clinic after a 7-week follow-up. The right eye’s visual acuity was 20/20 and the left eye was 20/25. Biomicroscopy revealed a significant reduction in epithelial crystal-like deposits and diffuse punctate epitheliopathy in both eyes (Figures 1 C, D). Hyperreflective intraepithelial deposits decreased significantly in IVCM imaging after cessation of BM (Figure 2E). There was a continued loss of the corneal subbasal corneal nerve plexus (Figure 2F). Corneal stroma and endothelium were within normal limits (Figures 2 G, H). Based on these findings, a reduced dosage of BM (1.9 mg/kg) was administered. There was no worsening of ocular toxicity during the follow-up period.

## DISCUSSION

Ocular side effects like blurred vision, dry eye, conjunctivitis, and uveitis are frequent and may occur during the treatment of different malignancies with various agents^(7)^. Corneal findings of BM include MECs, a hyper-reflective crystal-like appearance in the corneal epithelium and limbal stem cell deficiency. The mechanism of toxicity is still unknown, however, it is hypothesized that BM enters the cornea either via limbal vessels or tear film. Since the proteins are the main aim of ADC, like BCMA is not secreted by non-malignant cells, like corneal cells, the mechanism of the toxicity remains unknown^(8^, ^9^, ^10)^. According to Farooq et al. Fc-receptor- mediated endocytosis, pinocytosis, and by-stander toxicity are the presumed mechanisms^(9)^.

Since BM is a novel anti-myeloma agent, there is scarce available data in the literature about its ocular side effects. Bausell et al. identified MECs in all 12 patients, with MECs typically starting peripherally and expanding centrally with time^(8)^. It has been shown that the healing process also follows a centripetal pattern. When the diseased corneal epithelial cells appear centrally during this migration, visual acuity deteriorates. Some patients in both Bausell’s and Farooq’s series displayed a pattern of whorl-like fluorescein staining, which may indicate limbal stem cell deficiency^(8^,^9)^.

BM-containing cells are the source of hyperreflectivity in IVCM^(8^,^9)^. Roussea et al. presented a case with 3 diopters hyperopic shifts 1 week following the second infusion of BM^(10)^. The oblate topography was accompanied by microcystic opacities with hyperreflective dots in IVCM^(10)^. These opacities were especially noticeable in the wing cells. With the deferral of the subsequent infusion, all findings regressed. As demonstrated in the DREAMM-2 study group representative case, IVCM revealed intraepithelial and subepithelial hyperreflective deposits in wing and basal cells^(9)^.

Keratopathy and visual acuity scale for grading the toxicity designed for a standard follow-up to the corneal findings and visual acuity^(9)^. A detailed ophthalmological examination is suggested at the start of each infusion and whenever symptoms worsen. Dose or dosing interval adjustments are mandatory for reinitiating BM treatment.

This case report aimed to create awareness among ophthalmologists and hematologists about the frequent corneal toxicity of this novel ADC, BM, in myeloma patients.

## References

[1] Palumbo A, Anderson K (2011). Multiple myeloma. N Engl J Med.

[2] Gulla A, Anderson KC (2020). Multiple myeloma: the (r)evolution of current therapy and a
glance into future. Haematologica.

[3] Palumbo A, Avet-Loiseau H, Oliva S, Lokhorst HM, Goldschmidt H, Rosinol L (2015). Revised international staging system for multiple myeloma: a
report from International Myeloma Working Group. J Clin Oncol.

[4] Thorsteinsdottir S, Dickman PW, Landgren O, Blimark C, Hultcrantz M, Turesson I (2018). Dramatically improved survival in multiple myeloma patients in
the recent decade: results from a Swedish population-based
study. Haematologica.

[5] Markham A (2020). Belantamab Mafodotin: first approval. Drugs.

[6] Lonial S, Lee HC, Badros A, Trudel S, Nooka AK, Chari A (2020). Belantamab mafodotin for relapsed or refractory multiple myeloma
(DREAMM-2): a two-arm, randomised, open-label, phase 2 study. Lancet Oncol.

[7] Omoti AE, Omoti CE (2006). Ocular toxicity of systemic anticancer
chemotherapy. Pharm Pract (Granada).

[8] Bausell RB, Soleimani A, Vinnett A, Baroni MD, Staub SA, Binion K (2021). Corneal Changes After Belantamab Mafodotin in Multiple Myeloma
Patients. Eye Contact Lens.

[9] Farooq AV, Degli Esposti S, Popat R, Thulasi P, Lonial S, Nooka AK (2020). Corneal epithelial findings in patients with multiple myeloma
treated with Antibody-Drug Conjugate Belantamab Mafodotin in the Pivotal,
Randomized, DREAMM-2 Study. Ophthalmol Ther.

[10] Rousseau A, Michot JM, Labetoulle M (2020). Belantamab Mafotodin-Induced Epithelial Keratopathy Masquerading
Myopic Surgery. Ophthalmology.

